# Experimental and modeling study of ZnO:Ni nanoparticles for near-infrared light emitting diodes

**DOI:** 10.1039/d2ra00452f

**Published:** 2022-04-29

**Authors:** Imen Elhamdi, Hajer Souissi, Olfa Taktak, Jaber Elghoul, Souha Kammoun, Essebti Dhahri, Benilde F. O. Costa

**Affiliations:** Laboratoire de Physique Appliquée, Groupe de Physique des Matériaux Luminescents, Faculté des Sciences, Université de Sfax BP 1171 3000 Sfax Tunisia imen85356@gmail.com; Imam Mohammad Ibn Saud Islamic University (IMSIU), College of Sciences, Department of Physics Riyadh 11623 Saudi Arabia; Laboratory of Physics of Materials and Nanomaterials Applied at Environment (LaPhyMNE), Faculty of Sciences in Gabes, Gabes University 6072 Gabes Tunisia; University of Coimbra, CFisUC, Physics Department P-3004-516 Coimbra Portugal

## Abstract

This work is devoted to the synthesis and study of the different properties of ZnO nanoparticles (NPs) doped with the Ni element. We have used a simple co-precipitation technique for the synthesis of our samples and various structural, morphological and optical techniques for their analysis. Energy-Dispersive X-ray spectroscopy (EDX) confirms the stoichiometry of the samples. The X-Ray Diffraction (XRD) patterns reveal the hexagonal wurtzite phase of polycrystalline ZnO with a *P*63*mc* space group. Debye Scherrer and Williamson–Hall methods show that the average size of crystallites is around 40 nm. Transmission electron microscopy (TEM) images confirm the XRD results. The optical spectrum of Zn_0.95_Ni_0.5_O shows the presence of near-band-edge (NBE) ultraviolet emission. The absorption defect bands appearing near the blue–green region and near infrared emission are attributed to the Ni^2+^ intra-3d luminescence. The electronic structure of the Ni^2+^ doped ZnO NPs confirms the *T*_d_ site symmetry of Ni^2+^ in the ZnO host crystal and leads to a perfect correlation between calculated and experimental energy levels.

## Abstract 2

This work is devoted to the synthesis and study of the different properties of ZnO nanoparticles (NPs) doped with the Ni element.

## Graphical abstract

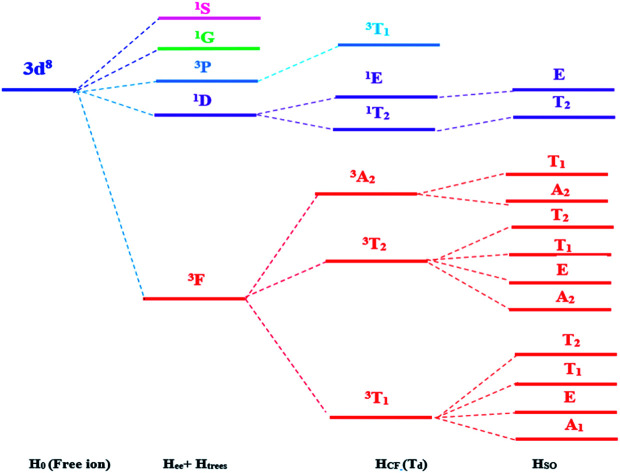

## 1 Introduction

ZnO is an attractive wide band gap II–VI semiconductor for potential applications in photonics, electronics, biosensors, biomedicine and spintronics.^1–10^ Due to good properties such as wide band gap (3.37 eV) and high excitation binding energy (60 meV), ZnO is among the useful materials for applications such as nanodevice fabrication, gas sensors, light emitting diodes, dye-sensitized solar cells, light emitting devices, thin film transistors, and optoelectronic devices.^10–18^ Likewise, it is widely used in various medicinal, cosmetic, and food products. Due to the anti-microbial and anti-tumor activities, ZnO is included in some cosmetic lotions as it is also known to maintain UV blocking and absorbing capabilities.^5^ ZnO can also be used as an astringent for wound healing, hemorrhoid treatment, eczema treatment and excoriation treatment in human medicine and veterinary science. ZnO nanoparticles (NPs) have also antibacterial, anti-neoplastic and antimicrobial properties.^5^ In order to modify the structure of the electronic band and improve its applications in various semiconductor spintronic devices, ZnO has been doped with different transition metals (TM).^6–18^ The Zn^2+^ in the crystal lattice structure of ZnO will be replaced by metal ions from doping, thus increasing the number of free electrons. This improves the electronic properties of the oxide semiconductor. ZnO NPs have been prepared by simple, low cost, solution-based methods, such as chemical precipitation, sol–gel synthesis, solvothermal and hydrothermal methods.^16–21^ The co-precipitation method is among the methods that have been widely used for the synthesis of NPs. It is considered to be promising method due to the ease of control of the particle size and the need for low temperatures in the synthesis process.

In the present work, the ZnO NPs doped with Nickel were synthesized by using chemical co-precipitation method. The structural, morphological and optical properties of the ZnO NPs doped with Ni^2+^ were studied. We present a detailed crystal field analysis of electron energy levels of Ni^2+^ doped ZnO NPs. A theoretical study, based on the Racah tensor algebra methods, was performed for the Ni^2+^ center (3d^8^) with *T*_d_ site symmetry.

## 2 Experimental methods

### 2.1 Materials

The precursors used to prepare ZnO:Ni^2+^ nanopowders, purchased from Sigma Aldrich, consist of high purity chemicals without further purification which are: zinc sulfate heptahydrate ZnSO_4_·7H_2_O (ACS reagent, 99%), nickel chloride hexahydrate NiCl_2_·6H_2_O (99.9%, trace metals basis), sodium hydroxide NaOH (reagent grade, ≥98%) and ethanol C_2_H_5_OH.

### 2.2 Synthesis of Ni-doped ZnO NPs

As it is known, the co-precipitation process does not require expensive and complex equipment and it is robust and reliable to control the shape and size of the particles.^18^ Therefore, we used this process to synthesize the Ni doped ZnO nanoparticles. The process consists of dissolving 5 g of zinc sulfate heptahydrate and 0.105 g of nickel chloride in 300 ml of ultra-pure water. Then, 50 ml of NaOH (10 M) was added dropwise to the solution which was stirred magnetically for 2 h at room temperature to produce a white gelatinous precipitate. Then we filtered and washed the white precipitates using ethanol and ultrapure water several times. This step was followed by drying for 4 h at 200 °C in a furnace and grinding in an agate mortar. Finally, the nanoparticles obtained were heat treated at 500 °C in air atmosphere for 2 h.

### 2.3 Characterization techniques

The crystal structure of the Ni doped ZnO NPs was analyzed on the basis of powder X-ray diffraction (XRD) data. The powder diffraction patterns were recorded with a STOE STADI P powder diffractometer in Debye–Scherrer geometry using Ge(111) monochromatic Cu-Kα1-radiation of wavelength *λ* = 1.5406 Å. The XRD measurements at room temperature were carried out in the locked coupled mode in the 2*θ* range from 10° to 80° with a step width of 0.02°. The surface morphology, size distribution and the composition of the elements of Ni-doped ZnO NPs were realized with Transmission Electron Microscopy TEM made on a JEOL 2011 JEOL JEM 2000 Ex microscope operating at accelerating voltage of 100 kV and probe current of ∼800 pA coupled to an Energy-Dispersive X-ray analysis (EDX) device. The micrograph patterns were recorded at 80 kV and the selected area electron diffraction (SAED) pattern was recorded by keeping camera constant 100 cm. The Fourier Transform Infrared (FTIR) was used to identify the elemental constituents of the material. The FTIR spectrum was recorded at room temperature in the range of 4000–400 cm^−1^ using Thermo Nicolet 380 with KBr pellet technique. An integrating sphere compatible with the powder samples attached to the UV-Vis-IR spectrophotometer (Shimadzu UV-3101 PC) has been used to measure of the optical absorbance in the wavelength range from 300 to 1800 nm. Room temperature photoluminescence (PL) spectra of our NPs have been carried using a JobinYvon-Horiba Triax 190 spectrometer with a 488 nm line of an argon ion laser and a spectral resolution of 0.3 nm, coupled with a liquid nitrogen-cooled CCD detector.

## 3 Theoretical crystal-field study

**Fig. 1:**
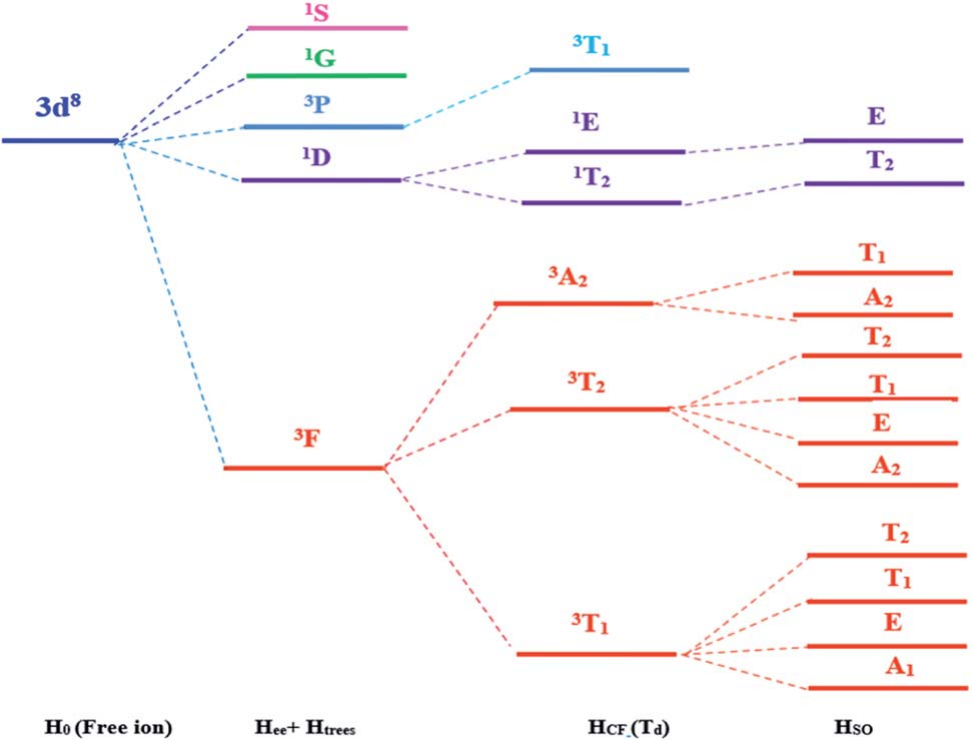
Energy levels of the Ni^2+^ ion (3d^8^) under the crystal field with *T*_d_ symmetry and spin orbit coupling.

When the free transition ion Ni^2+^ is used as a dopant of ZnO NPs, the energy levels are subdivided. The group theory allows us to predict the subdivision of the energy levels just by specifying the symmetry of the site occupied by the transition ion. This theory determines the number and the degeneracy of these energy levels and the value of these energies will be determined only by numerical calculation. The transition ion Ni^2+^ with 3d^8^ electronic configuration occupies a *T*_d_ site symmetry in ZnO. The decomposition of the *D*^(*L*)^ reducible representations of the SO(3) group (rotation group of the free transition ion) into irreducible representations of the site group *T*_d_ leads to the stark levels of the Ni^2+^ ions in the ZnO host matrix. Fig. 1 represents the splitting of the Ni^2+^ (3d^8^) energy level under the crystal field with *T*_d_ site symmetry. The energies of the stark levels (without and with the spin–orbit interaction) are obtained only by numerical calculation when using the Racah method. In order to study the energy levels splitting of Ni^2+^, we diagonalize the 45 × 45 matrix associated to the following Hamiltonian:^15–17,22–27^

1 *H* = *H*_0_ + *H*_ee_(*B*,*C*) + *H*_Trees_(*α*) + *H*_CF_(*D*_q_) + *H*_SO_(*ζ*)

*H*_0_ is the Hamiltonian configuration associated to the energy *E*_0_ which is 45 times degenerated. *H*_ee_ is associated to the electron–electron repulsions which leads to the Russell-Saunders terms: ^4^F (ground state according to Hund rule), ^4^P, ^2^H, ^2^G, ^2^F, ^2^D, ^2^D′ and ^2^P excited terms. The analytical eigenvalues of *H*_ee_ are a function of the Racah parameters *B* and *C*.^15^ The Trees correction Hamiltonian *H*_Trees_ represents the two-body orbit–orbit polarization interaction. The analytical eigenvalues of *H*_Trees_ are function of the Trees parameter *α*.^15–18,22^ For tetrahedral symmetry *T*_d_ and following the Wybourne's notation, the crystal field Hamiltonian *H*_CF_ is given by:^15–18,22–28^2
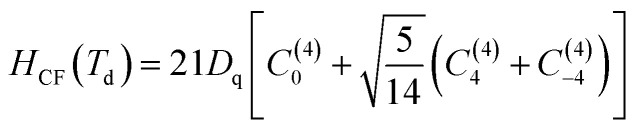


*D*_q_ is the crystal field strength parameter. *H*_CF_ is a function of the Racah tensor operators *C*_*q*_^(*k*)^, with:^15–18,22–28^3
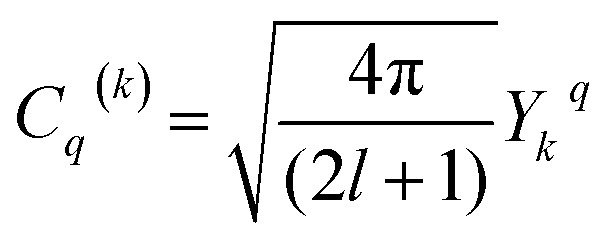


*Y*_*k*_^*q*^ are the spherical harmonics.

Using the Racah tensor algebraic methods and as we know that HCF is of the intermediate strength,^28^ we have calculated numerically the matrix elements of *C*_*q*_^(*k*)^ in the basis functions 
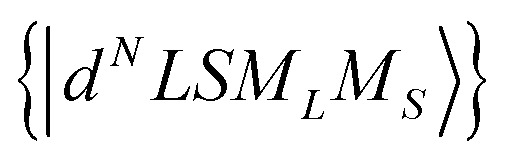
^15–18,22–28^4
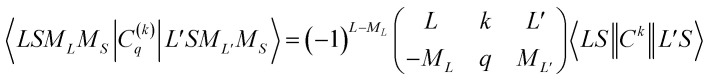



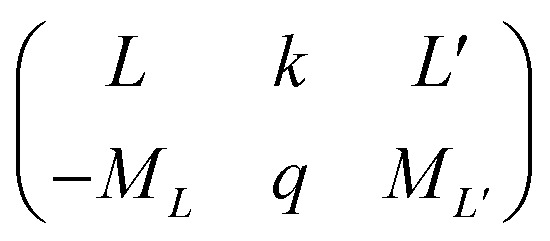
 is the 3*j*-symbols and 
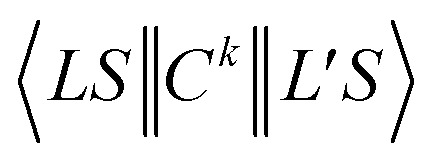
 is the reduced matrix elements.

The *B*, *C*, and *D*_q_ parameters are deduced from the optical spectra. The eigenvalue of the spin–orbit coupling Hamiltonian H_SO_ depends on *ξ* parameter.

The free ion Ni^2+^ parameters are: *B*_0_ = 1041 cm^−1^, *C*_0_ = 4831 cm^−1^, *α*_0_ = 42 cm^−1^ and *ζ*_0_ = 702 cm^−1^.^16,17,22,29–31^

An approximation is used between the parameters of doped and the free Ni^2+^ ion:^26,27^

5 *B* = *N*^4^*B*_0_, *C* = *N*^4^*C*_0_, *α* = *N*^4^*α*_0_ and *ζ* = *N*^2^*ζ*_0_

The average reduction factor *N* is defined as:^26,27^6
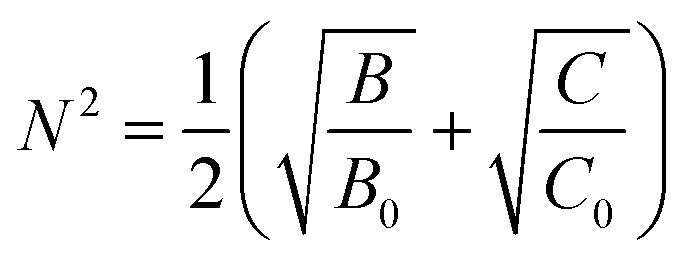


We created a Maple software code to determine the electronic structure of a transition ion having 3d^*n*^ electronic configurations and occupying a site with symmetry determined by one of the 32-point crystallographic groups. The ref. 26 and 27 provide more information about this code. This code produces the same results as Yeung and Rudowicz's code.^32^

## 4 Results and discussion

### 4.1 Energy-dispersive X-ray spectroscopy analysis (EDX)

**Fig. 2:**
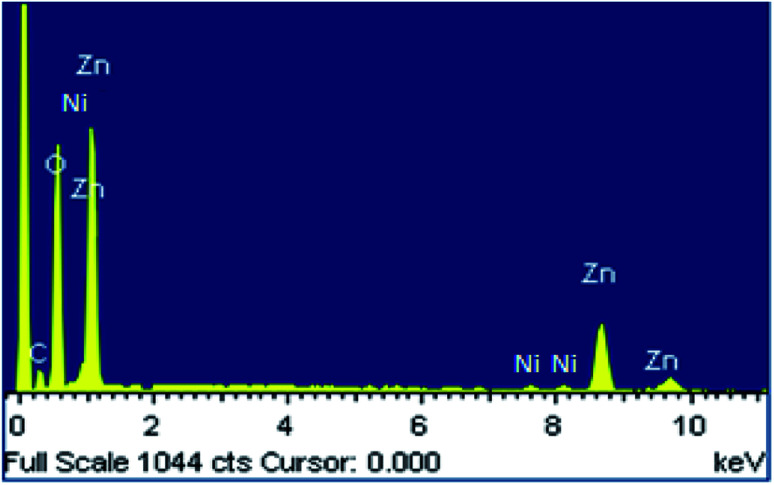
EDX spectrum of Ni doped ZnO NPs.

The EDX spectrum of the prepared ZnO NPs doped with Ni^2+^ is shown in Fig. 2. We know that peak C in the EDX spectrum is linked to the emission of the carbon ribbon used during the measurement. We note the presence of Zn and O as main components with low levels of Ni. This confirms the correct insertion of the doping element during preparation. In addition, there is no trace of peaks of external impurities in the studied compound confirming the purity of this material. The observation of low intensity peak for nickel doped ZnO in Fig. 2, confirms the proper incorporation of nickel Ni^2+^ in the ZnO structure. The quantitative analysis of the compositional elements presents in Ni-doped ZnO NPs using EDX spectra is presented in Table 1. From this analysis, the average Ni/Zn atomic percentage ratio is derived to be 5.01% in Ni-doped ZnO samples. This result confirms the good agreement with the experimental concentration used during the synthesis.

**Table:** The quantitative analysis of the compositional elements presents in Ni-doped ZnO NPs using EDX

Spectrum for Zn_0.95_Ni_0.05_O	O	Ni	Zn
Weight (%)	39.08	2.75	58.17
Ni/Zn ratio		0.0472	
Atomic (%)	72.42	1.31	26.27
Ni/Zn ratio		0.0498	

### 4.2 XRD diffraction

**Fig. 3:**
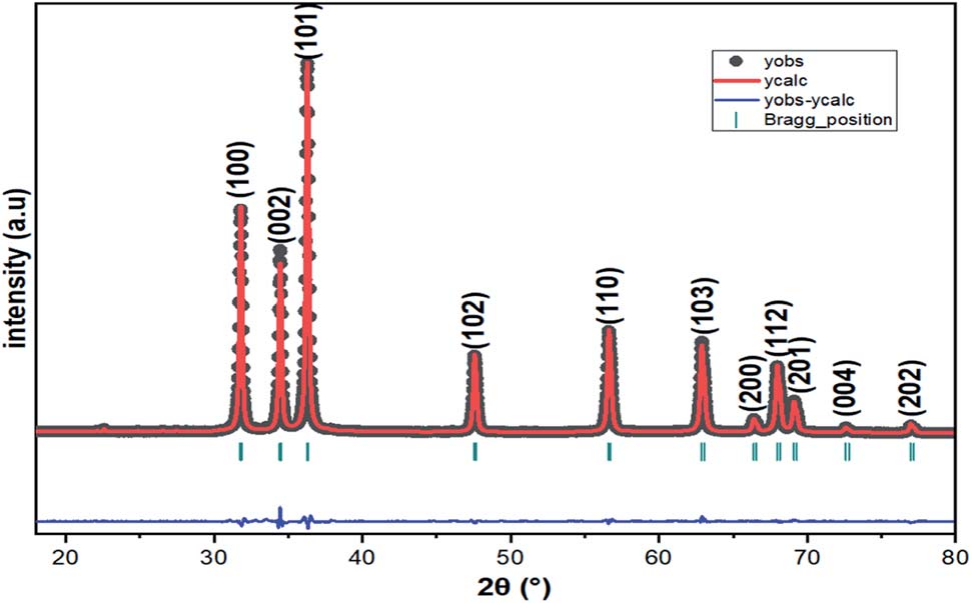
Powder X-ray diffraction pattern of Zn_0.95_Ni_0.05_O NPs.

**Fig. 4:**
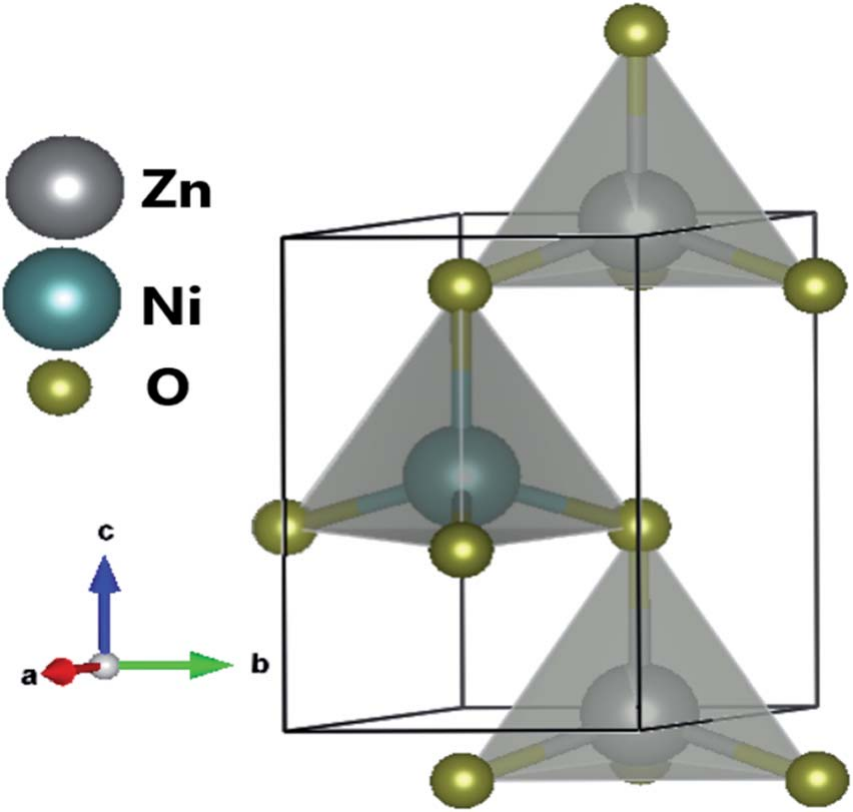
Crystal structure of ZnO doped with Ni^2+^.

The XRD pattern of Fig. 3 clearly shows a good crystallinity of prepared samples Zn_0.95_Ni_0.05_O NPs. The XRD peaks, located at 2*θ* values of 31.786° (100), 34.4256° (002), 36.2574° (101), 47.5444° (102), 56.5858° (110), 62.8694° (103), 66.3757° (200), 67.9516° (112) and 69.0743° (201), have been indexed using the standard ICDD file No. 36-1451 for ZnO.^33^ This XRD analysis confirms the single phase with a hexagonal wurtzite structure of ZnO lattice (space group *P*6_3*mc*_). It is noted that the wurtzite structure is not affected by the Ni transition ion doping. The Zn_0.95_Ni_0.05_O diffraction peaks show a small shift towards higher 2*θ* angle values compared to undoped ZnO NPs. In addition, the intensity of these peaks has decreased and the line widths broadened for the Zn_0.95_Ni_0.05_O NPs compared to the undoped ZnO NPs. The increase in lattice disorder and the deformation induced by the Ni^2+^ substitution can explain the change in XRD pattern. The ZnO diffraction peak (101) is the most intense peak indicating that the crystallographic preferential orientation is according to (101). In this structure, the Ni^2+^ ion replaces the Zn^2+^ ion, by substitution, in the ZnO structure and occupies a tetrahedral Td site symmetrically surrounded by four oxygen atoms with a slight distortion (Fig. 4).^11,12^

Interplanar distance between the planes of given Miller indices *h*, *k* and *l* called *d*_*hkl*_ as a function of the lattice constants (*a*, *c*) is given by the following relation:^19,20^7
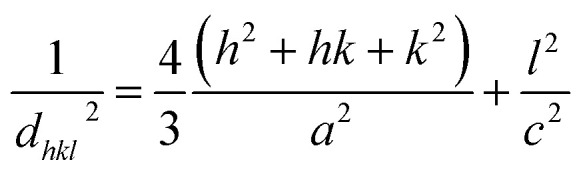


The plane spacing *d*_*hkl*_ satisfies the Bragg's law:^9^

8 *nλ* = 2*d*_*hkl*_ sin *θ*

*N* is the order of diffraction (usually *n* = 1), *λ* is the X-ray wavelength.

The lattice parameters *a*, *b* and *c* for Zn_0.95_Ni_0.05_O NPs are calculated from eqn (1) and (2) (*a* = *b* = 3.2497 Å and *c* = 5.2086 Å). The lattice parameters for undoped hexagonal wurtzite ZnO are *a* = *b* = 3.2513 Å and *c* = 5.2092 Å.^21^ The XRD refinements were also performed by Rietveld refinement software (Fullprof). The calculated pattern is consistent with the observed data and gives the same results for the lattice parameters *a*, *b* and *c* (Fig. 2). The refined crystallographic parameters are given in Table 2. The values of the parameters a and *c* of the Zn_0.95_Ni_0.05_O NPs are smaller compared to those of the undoped ZnO NPs, which is related to the fact that the ionic rays of the dopant Ni^+2^ (0.69 Å) is lower than the ionic radii of the substituted Zn^+2^ (0.74 Å). The value of interplanar spacing *d*_101_(2.4760 Å) for Zn_0.95_Ni_0.05_O calculated from XRD data for (101) plan shows a slightly decrease compared to *d*_101_ (2.4770 Å) for undoped ZnO. This behavior is surely due to the variation in bond lengths and angles between atoms caused by the distortion of the NiO_4_ tetrahedron, causing lattice stress. The tensile stress causes the peak to shift to lower 2*θ* values due to the increased spacing *d*, whereas the compressive stress plays an opposite role.

Results with Fullprof refinement

**Table:** 

*R*_p_	*R*_wp_	*R*_exp_	*χ* ^2^
4.75	6.13	2.49	6.05

**Table:** 

*a* (Å)	*b* (Å)	*c* (Å)	*α* (°)	*β* (°)	*γ* (°)
3.249_7_	3.2497	5.2057	90.0000	90.0000	120.0000

**Table:** 

Atom	*X*	*Y*	*Z*	Occupation
Zn	0.3333	0.6667	0.0000	0.9254
Ni	0.3333	0.6667	0.0000	0.0254
O	0.3333	0.6667	0.3833	1.0010

In the direction of *c* axis, the Zn–O bond length *L* has been calculated using the following formula:^19,20^9
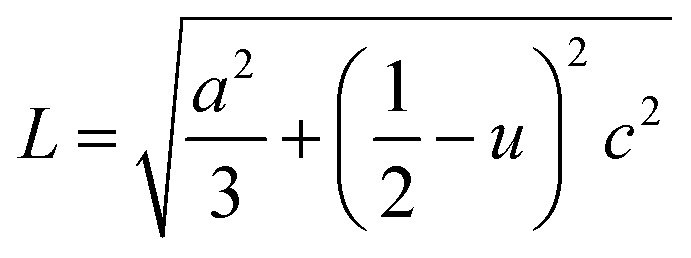


The positional parameter *u* in the wurtzite structure measures the amount which each atom gets displaced with respect to the next.^33,34^ The parameter *u* is determined by the relation:10
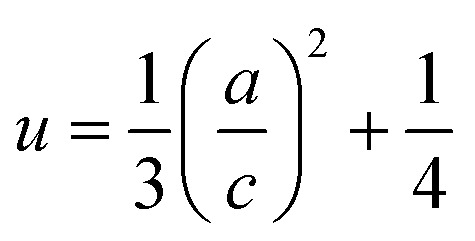


The value of *u* is of 0.375 when the network is ideal. The variation of *u* indicates a modification of the lattice parameters. The calculated *L* is of 1.9779 for Zn_0.95_Ni_0.05_O (*L* is of 1.9787 for undoped ZnO). A good agreement between *L* of this study and with those reported in literature^21^ is obtained which supports the results of the present study.

The volume of the unit cell for the hexagonal system has been calculated using the following relation:^19^11
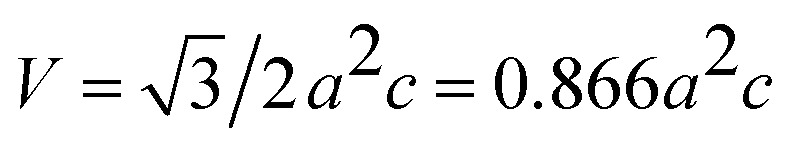


The volume of the unit cell is of 47.6349 Å^3^.

#### 4.2.1 Debye–Scherrer method

The crystallite size *D* of Zn_0.95_Ni_0.05_O nanoparticles was calculated from the Debye–Scherrer's formula from XRD pattern. This formula is given by the following expression:^35^12
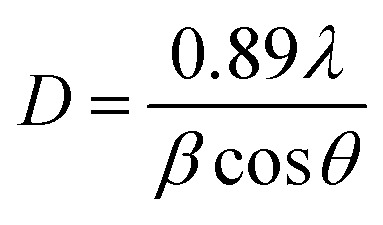


The incident X-ray radiation wavelength *λ* is of 0.15418 nm. The Bragg angle *θ* represents the position of the diffraction peak in the diffractograms. *β* is expressed in radian and represents the full width at half maxima (FWHM) of X-ray diffraction peak. The crystallite size *D* is calculated from the intense XRD peak and was found to be 36.06 nm.

The density of dislocation (*δ*) measures the defects concentration in nanostructures. The *δ* is considered as the ratio of the dislocation length and volume of the crystal and is calculated using the equation:^36^

13 *δ* = 1/*D*^2^

The crystallite size *D* is calculated above from eqn (6). The calculated value of dislocation density (*δ*) for synthesized Zn_0.95_Ni_0.05_O is of 7.6870 × 10^−4^ nm^−2^.

#### 4.2.2 Williamson–Hall method

The broadening of the DRX peaks arises not only from the crystallites size effect, but also from the stress in the particles. The Williamson–Hall method makes it possible to calculate the size of the crystallites *D* and also the value of the micro deformation *ε*. The eqn (8) represents the Williamson–Hall model modified, which manifests the Uniform Deformation Model (UDM):^37^

14 *β* cos *θ* = 0.89*λ*/D + 4*ε* sin *θ*

*λ* is the wavelength of incident X-ray radiation, *β* is expressed in radian and represents the full width at half maxima (FWHM) of the peaks of X-ray patterns.

**Fig. 5:**
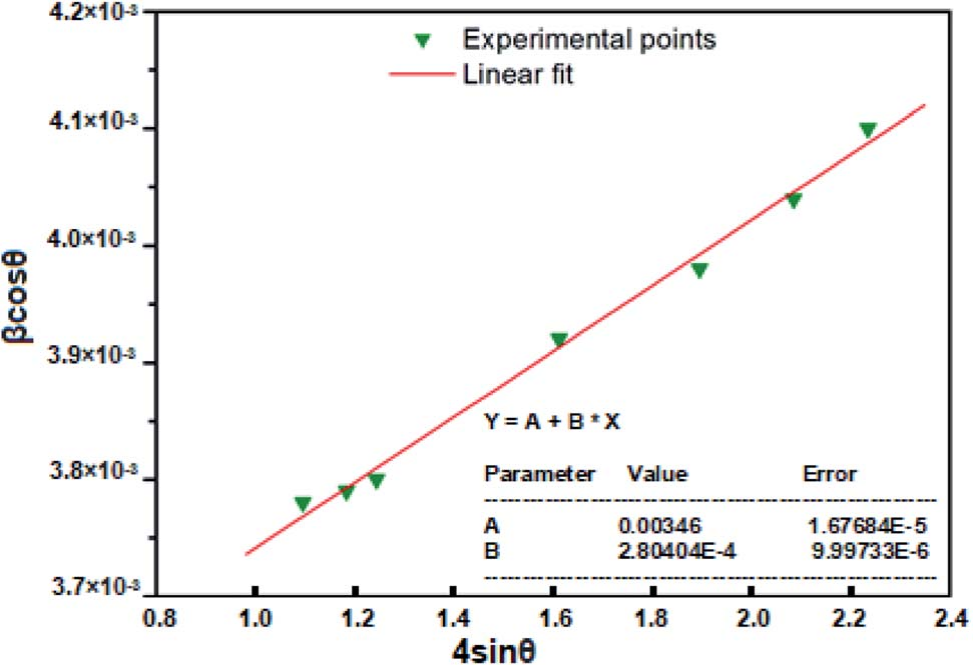
The W–H analysis of Zn_0.95_Ni_0.05_O assuming UDM model.

By using Origin 2018, we have plotted (*β* cos *θ*) as a function of (4 sin *θ*) for the preferred orientation peaks in the XRD pattern (following peaks (100), (002), (101), (102), (110), (103) and (112)) of Zn_0.95_Ni_0.05_O NPs with the wurtzite hexagonal phase. From the obtained Williamson–Hall plots (Fig. 5), the crystallite size and the strain deduced from the intersection *y*-axis and slope of the linear fit was 39.6 nm and 2.8040 × 10^−4^, respectively. The crystallite size evaluated by the Williamson–Hall model is larger than that obtained by the Scherrer model. This is due to the fact that the effect of broadening of the X-ray peaks of Fig. 3, results from the size of the crystallites and the presence of deformation, and this latter is completely neglected in the Scherrer method. The strain *ε* obtained from the Williamson–Hall model is an important factor to consider and has a remarkable effect on the size of the crystallites.

### 4.3 Transmission electron microscope (TEM) studies

**Fig. 6:**
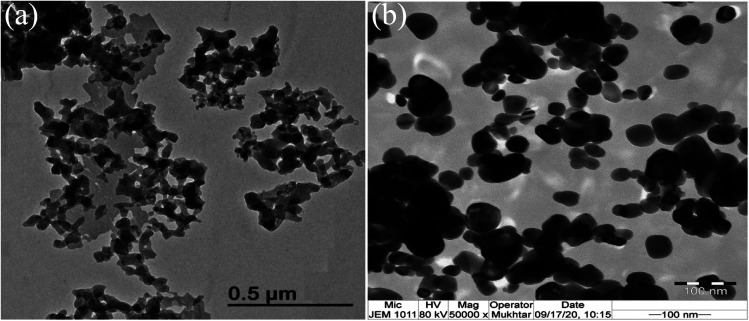
TEM image of Ni doped ZnO NPs (a) around 500 nm and (b) around 100 nm.

**Fig. 7:**
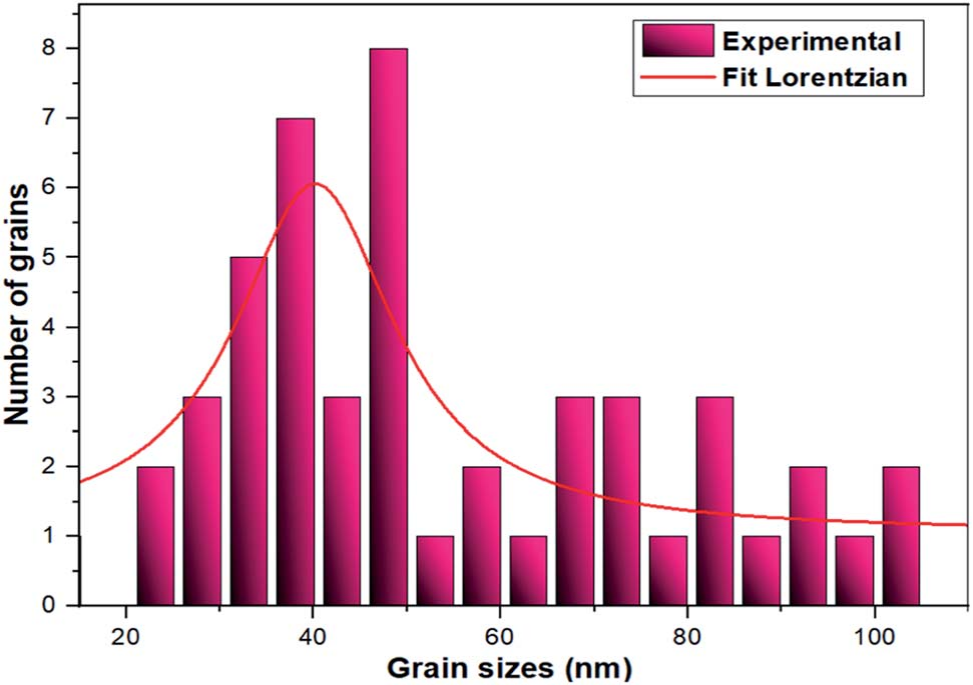
Histogram of grain size distribution of Ni-doped ZnO NPs.

**Fig. 8:**
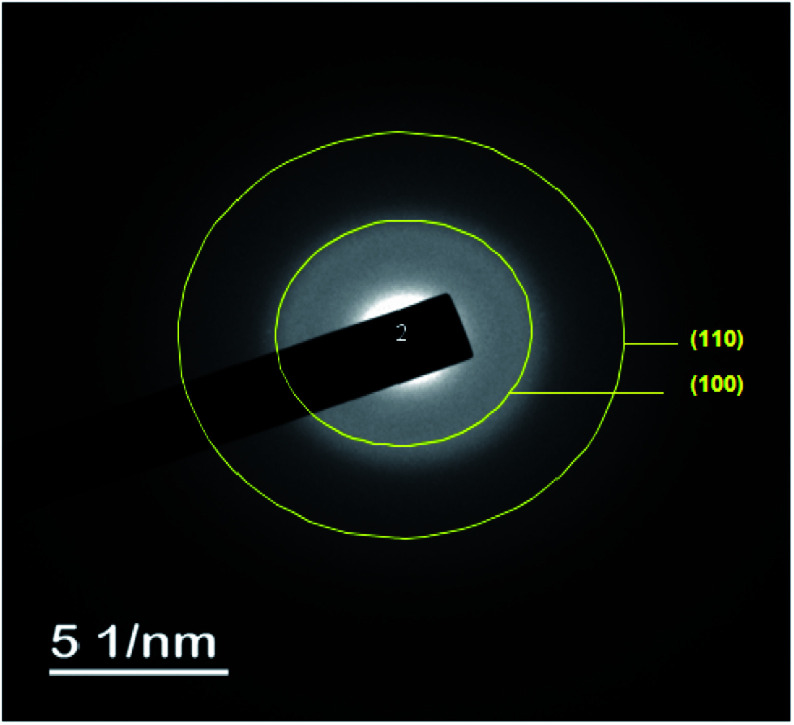
The selected area electron diffraction (SAED) image Ni doped ZnO NPs.

The TEM observations are is a good tool for the morphological studies of ZnO NPs and to determine the particle size and shape. TEM micrographs of Zn_0.95_Ni_0.05_O NPs, presented in Fig. 6a and b, indicate the formation of nanometric particles with a well-confined but agglomerated structure. When compared to undoped ZnO, the nickel doped ZnO samples have a random hexagonal and spherical shape with lower particle sizes. The distribution of particles is heterogeneous and the particles appear to be broadly agglomerated. Because of the increased surface to volume ratio, which results in an increase in attractive force, the particles tend to agglomerate together. This is due to nickel incorporation in the ZnO lattice. The Fig. 7 shows the particle size distribution histogram of Zn_0.95_Ni_0.05_O NPs. The particle size spans from 20 to 100 nm on the histogram, with an average size of 40.173 nm. This average value is equivalent to the estimated crystallite size calculated by Willamson–Hall method. The SAED image of Zn_0.95_Ni_0.05_O system, shown in Fig. 8, reveals a ring pattern confirming the polycrystalline structure of the sample. The indexing of SAED pattern matches well with XRD data.

### 4.4 Fourier transform infrared (FTIR) studies

**Fig. 9:**
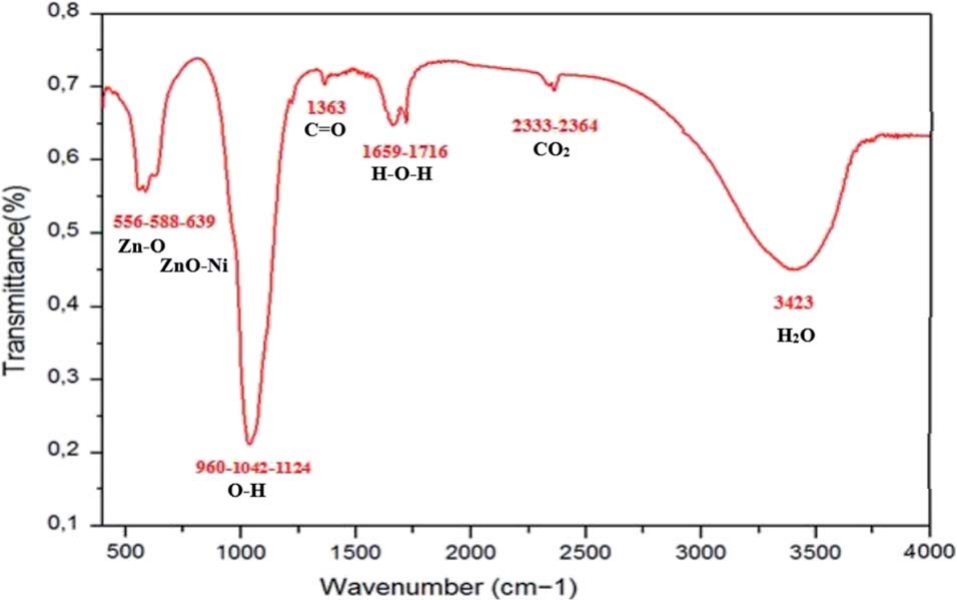
FTIR spectra of Ni-doped ZnO NPs at room temperature.

The FTIR spectrum for Zn_0.95_Ni_0.05_ NPs was recorded in the range of 400–4000 cm^−1^ and is shown in Fig. 9. The FTIR spectrum provides further information on functional groups. The presence and the position of the absorption bands depend on the crystal structure, chemical composition and particle morphology. FTIR measurements confirmed the creation of the wurtzite structure in Ni doped ZnO. Table 3 lists the persistent IR frequencies, as well as the vibrational assignments, that are responsible for Ni-doping in ZnO at room temperature. In the hexagonal wurtzite type crystal structure, FTIR spectra reveal the existence of characteristic peaks in the ranges of 450 to 650 cm^−1^, which correspond to Zn–O stretching vibrational modes.^38^ The change in bond lengths of Zn–O lattice when Ni ion replaces Zn ion leads to a shift of the vibration frequency of Zn–O to a frequency higher than 434 cm^−1^ corresponding to undoped ZnO. This confirms the incorporation of Ni^2+^ ions as a substitute of Zn^2+^ ions in ZnO structure. The vibrations of ZnO–Ni local bonds and defect states are responsible for the absorption bands that emerge about 640 and 960 cm^−1^, respectively.^38^ From literature, these frequency are absent in undoped ZnO sample. The bands around 1042 cm^−1^ are strong with asymmetric stretching of resonance interaction between vibration modes of oxide ions in the nanocrystals. The asymmetric and symmetric stretching of the carboxyl group (C=O) correlate to the absorption peaks seen at 1350 and 1400 cm^−1^. The peak around 1659 cm^−1^ in ZnO is ascribed to H–O–H bending vibration and is assigned to a little amount of H_2_O in the ZnO NPs. The absorption peaks observed in the range 2320–2400 cm^−1^are due to the existence of CO_2_ molecule in air. The broad absorption peak (2700–3500 cm^−1^) centered at 3443 cm^−1^ is ascribed to normal polymeric O–H stretching vibration of H_2_O molecule in Zn–O lattice.^38^ The appearance of these bands in manufactured nanoparticles could be attributed to atmospheric water content adsorption.

**Table:** FTIR results for ZnO doped with Ni

Assignments	Wavenumber (cm^−1^)
Zn–O stretching mode	450–650
Vibrations of ZnO–Ni local bonds	640
Defect states	960
O–H asymmetric stretching	1042
Symmetric stretching of the carboxyl group (C=O)	1350–1400
H–O–H bending vibration	1659
CO_2_ molecule in air	2320–2400
O–H stretching vibration of H_2_O	3443

### 4.5 Investigation of the optical spectra

**Fig. 10:**
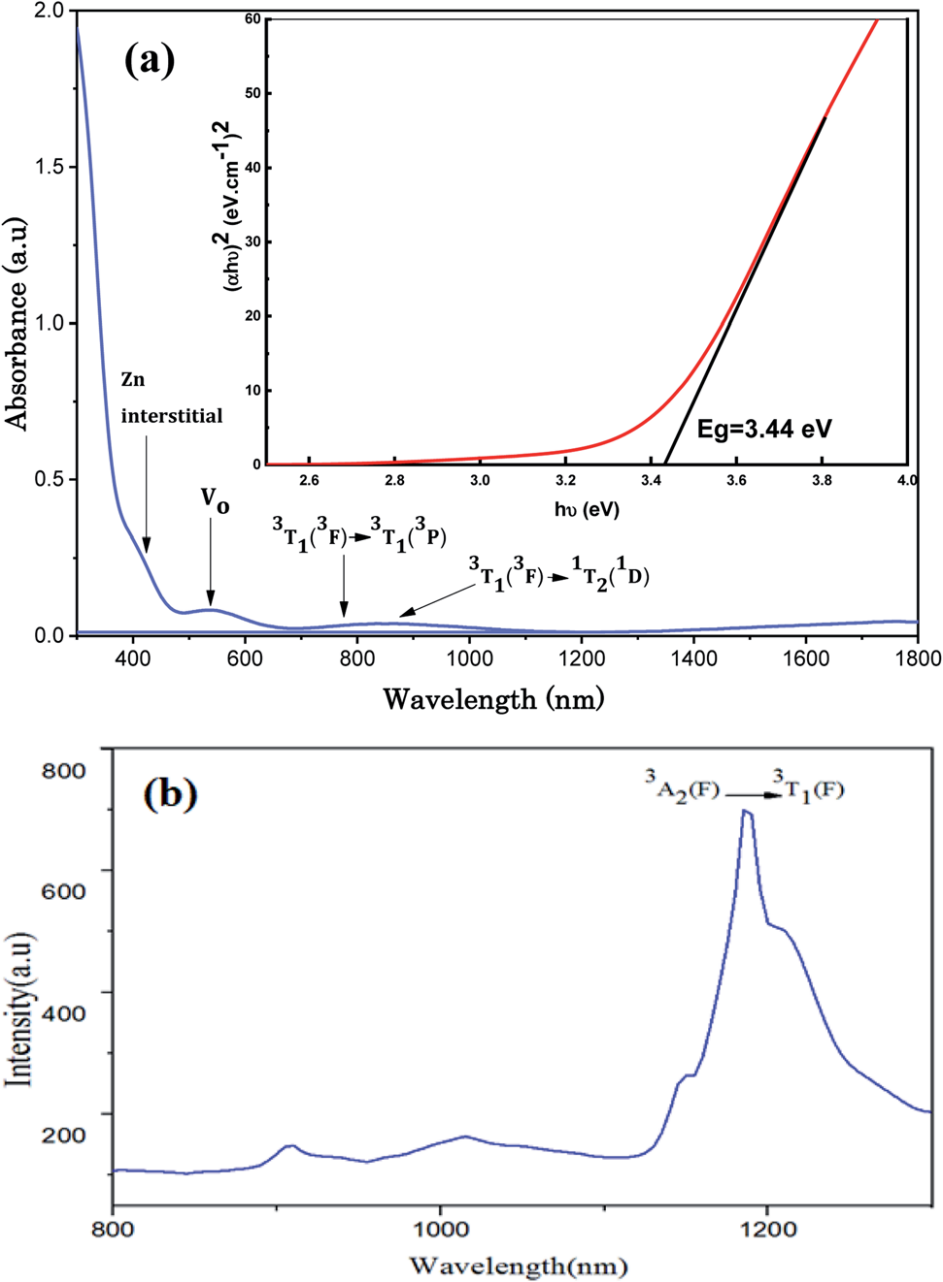
(a) Absorption spectrum (300–1800 nm) and (b) PL spectrum (800–1300 nm).

The UV-Visible optical absorption spectrum of Zn_0.95_Ni_0.05_O NPs recorded at room temperature (300–1800 nm) and the PL spectrum (800–1300 nm) are illustrated in Fig. 10.

#### 4.5.1 The optical band gap *E*_g_

The optical band gap *E*_g_ of Zn_0.95_Ni_0.05_O semiconductor was calculated using the Tauc's law given by:^39–41^

15 *αhν* = *A*(*hν* − *E*_g_)^*n*^

where *hν* is the energy of the incident photon, *α* is the absorption co-efficient and *A* is a constant. The type of transition depends on the exponent *n* which can take two values depending on whether the transition is allowed direct (*n* = 1/2) or indirect band gaps (*n* = 2). From Fig. 10a, the optical transition is direct and the energy band gap *E*_g_ of Zn_0.95_Ni_0.05_O is of 3.44 eV. The value of *E*_g_ indicates that the NPs Zn_0.95_Ni_0.05_O are characterized by a wide band-gap material useful in opto-electronic devices. We remark that the values of the *E*_g_ of the Zn_0.95_Ni_0.05_O NPs semiconductors decrease compared to bulk ZnO for which the *E*_g_ is 3.37 eV.^16,17^

#### 4.5.2 defects in ZnO

Several recent works have contributed to the search for intrinsic point defects in ZnO nanocrystals.^42–48^ Shankari Nadupalli *et al.* uses electron paramagnetic resonance (EPR) and photoluminescence (PL) spectroscopy to analyze the intrinsic defect structure of ZnO. They compare the findings to previous computational work. The defects are analyzed primarily by considering the microscopic defect structure of the lattice. Intrinsic defects in ZnO modify its electrical and optical properties. Shankari Nadupalli *et al.* briefly reviews the existing information on the local structure surrounding the defects.^42^

Defects commonly mentioned in ZnO-based nanostructures are:^42–48^

- Oxygen vacancies with different charged states (the +2 charged state (V_O_^2+^), the +1 charged state (V_O_^+^) and the neutral state (V_O_^0^)); the resulting emissions related to this type of defect usually occur near blue–green (approximately 420–550 nm).

- Zn vacancies; the resulting emissions related to this type of defect usually occur near yellow (approximately 550–610 nm).

- Zn interstitials; in the wurtzite ZnO structure, Zn_*i*_ occupies the tetrahedral or the octahedral site. When compared to the interstitial occupying tetrahedral site, the octahedral occupancy of Zn_*i*_ is shown to be a stable and favorable state.^17^ It has been demonstrated that The Co^2+^ implantation generates a high concentration of additional zinc interstitials as it substitutes the Zn ions.^17^ This confirms that the intense donor bound exciton emission at 3.36 eV at low temperatures assigned to the D°X line is related to zinc interstitials Zn_*i*_.^17^ We assume in this work the same behavior with the incorporation of the transition ion Ni^2+^. The resulting emissions related to this type of defect usually occur near orange-red (approximately 610–750 nm).

The absorption peak at 430 nm may be due to the electron transition from the valence band to Zn interstitial energy level. The absorption in the green region (511–600 nm) is related to deep level absorption (DLE). VO related defects and V_Zn_ contribute to this broad band absorption. The most common defects in ZnO are oxygen vacancies and generally act as radiative centers in luminescence processes. These oxygen vacancies are considered among the intrinsic defects of n-type ZnO. By capturing electrons they generate a new level of energy that can form ionized vacancies which act as deep defect donors. These deep defects further influence the optical properties of ZnO. The green absorption located at 534 nm is certainly related to the oxygen vacancies.

**Fig. 11:**
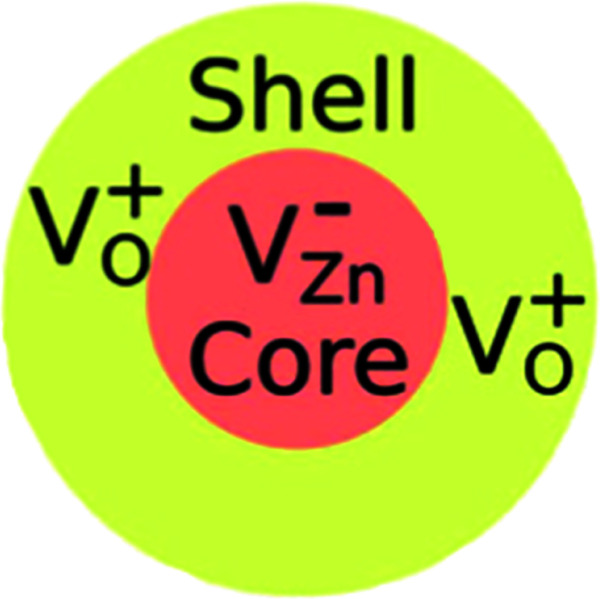
The surface/shell defects is attributed to an unpaired electron-trapped oxygen vacancy (V_O_) site and the core defects is attributed to V_Zn_^−^ centers [Shankari Nadupalli].

Electron paramagnetic resonance (EPR) spectroscopy is one approach for properly observing and distinguishing defects in ZnO crystals.^42,46,48^ According to Shankari Nadupalli *et al.*, two EPR signals are observed for ZnO nanocrystals at *g* = 1.9620 and *g* = 2.0048, depending on the size and shape of the ZnO particle. These findings suggest that the surface/volume ratio has an impact on the sort of defects incorporated in ZnO. By using the core–shell model, core defects are assigned to EPR lines at *g* = 1.9620, whereas surface/shell defects are attributed to EPR lines at *g* = 2.0048 (Fig. 11). The EPR signal of surface/shell defects is attributed to an unpaired electron-trapped oxygen vacancy (*V*_O_) site and can either be *V*_O_^+^ or V_O_^0^, depending on the size and morphology of ZnO. Whereas, the EPR signal of the core defects is attributed to V_Zn_^−^ defect centers^42^ (Fig. 11). This result is specifically for ZnO nanocrystals. According to this concept and by tuning the size of the ZnO nanocrystal, one can achieve a negatively charged (n-type) core with a positively charged (p-type) surface or an exclusive p-type ZnO quantum dot. The core–shell model is only applicable to nanosized ZnO particles and cannot be used to simulate microcrystalline (>500 nm) or bulk ZnO samples.^42,46,48^ The research presented by Shankari Nadupalli *et al.* predicted that changing the size of the nanocrystal can change the concentration of intrinsic defects and their location.^42^ The size of the nanocrystal and the post-annealing treatments can also affect the UV, green, orange, and red emissions.^42^ From ref. 42, the calculated energy level for Zn_*i*_ is 0.22 eV below the conduction band and V_Zn_ is 0.30 eV above the valence band. The transition Zn_*i*_ → V_Zn_ leads to a radiative emission of 2.98 eV (415 nm). The defect level of *V*_O_ is located at 0.9 eV above the valence band. Then, the transition between Zn_*i*_ → *V*_O_ results in a radiative emission of 2.38 eV (520 nm).^42^

#### 4.5.3 The determination of crystal field *D*_q_, Racah B, C, Trees *α* and spin–orbit parameters from absorption spectra

The optical spectrum of Zn_0.95_Ni_0.05_O NPs in the visible region (Fig. 10a and b) show three bands located at:

- 763 nm (13106 cm^−1^) on the absorption spectrum corresponding to the spin allowed transition ^3^T_1_ (^3^F) → ^3^T_1_ (^3^P).

- 859 nm (11641 cm^−1^) on the absorption spectrum corresponding to the spin forbidden transition ^3^T_1_ (^3^F) → ^1^T_2_ (^1^D).

- 1187 nm (8424 cm^−1^) on the emission spectrum corresponding to the spin allowed transition ^3^A_2_ (^3^F) → ^3^T_1_ (^3^F).

**Fig. 12:**
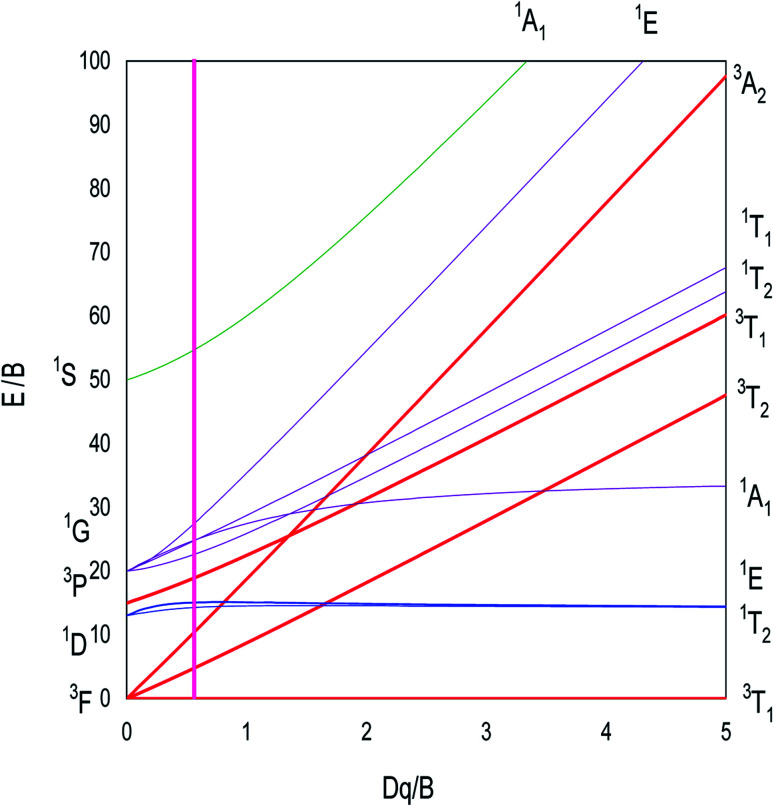
Tanabe–Sugano diagram for tetrahedral coordinated Ni^2+^ ion with *C*/*B* = 4.64. The vertical line with *D*_q_/*B* = 0.57 represents the case of ZnO:Ni^2+^ NPs.

The analytical expressions of the Ni^2+^ energies are obtained by calculating the eigenvalues of the Hamiltonian (45 × 45) of eqn (1). The obtained eigenvalues are a function of the average reduction factor *N* and the crystal field strength *D*_q_. These two parameters have been determined from the observed energies of the absorbance spectra of Zn_0.95_Ni_0.05_O NPs recorded at room temperature. We consider the ratio *C*/*B* = 4.64 of the free ion. The *B*, *C*, *α* and *ξ*_d_ parameters are calculated from eqn (5). All the parameters *N*, *D*_q_, *B*, *C*, *α* and *ξ*_d_ are listed in Table 4. The calculated parameters lead to the theoretical energy levels of ZnO NPs doped with Ni^2+^ with the use of the code developed by S. Kammoun *et al.* (Table 5). The reports: *β*_B_ = *B*/*B*_0_ = 0.71 and *β*_C_ = *C*/*C*_0_ = 0.71 make clear the covalency effects. The Tanabe–Sugano diagram, schematized in Fig. 12 for Ni^2+^ ion (*C*/*B* = 4.64), indicates the suitable value for (*D*q/*B* = 0.57) by the vertical line obtained from this theoretical analysis.

**Table:** The parameters values of *D*_q_, *N*, *B*, *C*, *α* and *ξ*_d_ (in cm^−1^) for Ni^2+^:ZnO of this work and comparison with literature

Materials/parameters	*D*_q_	*N* ^2^	*B*	*C*	*α*	*ξ*_d_
ZnO:Ni^2+^ nanoparticles [this work]	420	0.851	740	3433	30.458	597.8
Ni^2+^ in ZnO^35^	405		795	—		
Zn_2_SiO_4_:Ni^2+^ (ref. 36)	450		780	3630		

**Table:** Experimental and calculated energies (cm^−1^) of Ni^2+^ occupying a *T*_d_ symmetry in ZnO NPs

*T*_d_	*E*_observed_ (cm^−1^)	*E*_calculated_ (cm^−1^) *T*_d_ site symmetry	*T*′_d_	*E*′_calculated_ (cm^−1^)
^3^T_1_(^3^F)	0	0	A_1_	0 (1)
			T_2_	140 (3)
			T_1_	1002 (3)
			E	1535 (2)
^3^T_2_(^3^F)		3564	A_2_	3933 (1)
			T_1_	4008 (3)
			E	4478 (2)
			T_2_	4606 (3)
^3^A_2_(^3^F)	8424	7764	T_1_	8592 (3)
^1^T_2_(^1^D)	11641	11527 (3)	T_2_	
^1^E(^1^D)		12111 (2)	^1^A_1_	
^3^T_1_(^3^P)	13106	14028	A_1_	13824 (1)
			T_2_	14110 (3)
			T_1_	14685 (3)
			E	14686 (2)
^1^T_1_(^1^G)		17712 (3)		
^1^T_2_(^1^G)		19310 (3)	T_2_	
^1^A_1_(^1^G)		19432 (1)	E	
^1^E(^1^G)		21328 (2)	T_1_	
^1^A_1_(^1^S)		43752 (1)	A_1_	

#### 4.5.4 Photoluminescence (PL) studies

**Fig. 13:**
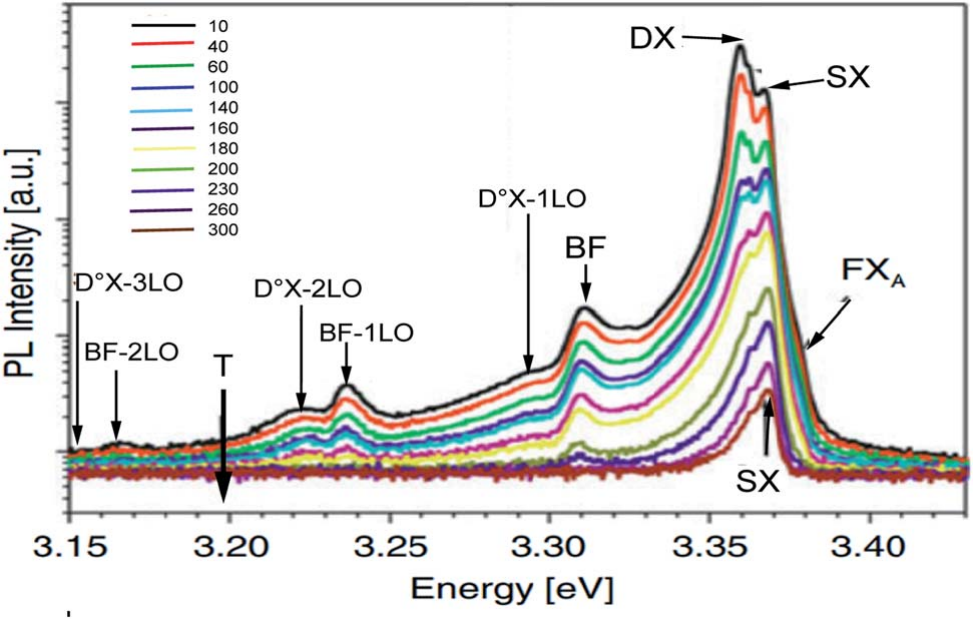
The PL spectra of Ni doped ZnO NPs at temperatures between 10 and 300 K.

**Fig. 14:**
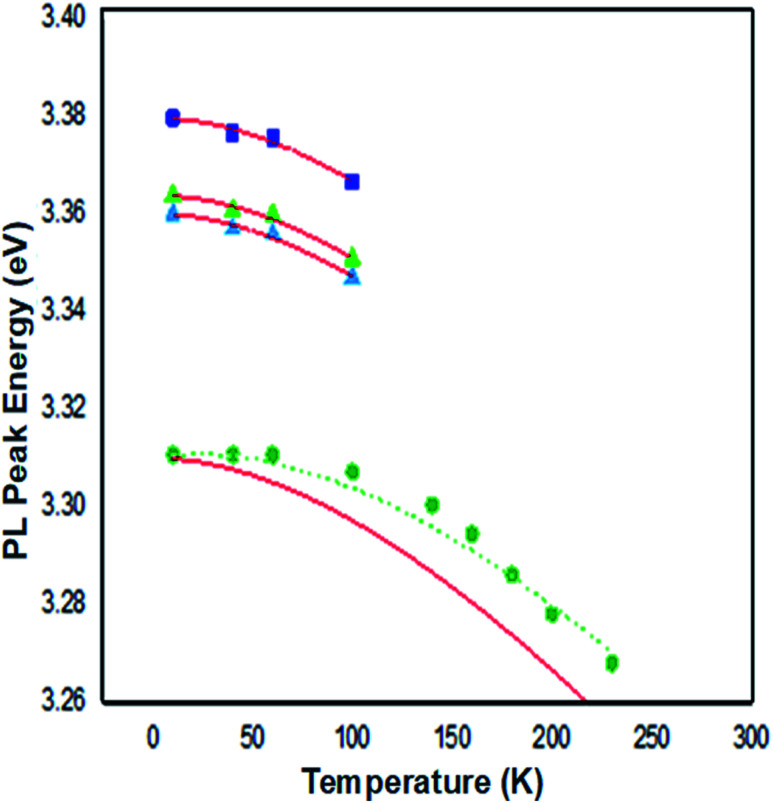
The temperature dependence of the peak energies for the FX_A_ emission (solid squares), the D°X emission (solid triangles), BF emission (reverse solid triangles) and the BF-1LO emission (solid circles). Solid lines are calculated using Varshni's formula. The dotted line is calculated using the formula *hν* = *E*_g_ − *E*_D_ + *k*_B_*T*.

The photoluminescence PL spectra of Zn_0.95_Ni_0.5_O represent the ultraviolet (UV) near-band-edge emission (3.15–3.43 eV) (Fig. 13). On the high-energy side of the PL spectra, the shoulder at 3.3795 eV appears at 10 K and is present for all temperatures. With increasing temperature, the shoulder's position moves to the low-energy side and is maintained until around 230 K. This shoulder is attributed to radiative recombination of free excitons FX_A_. The recombination at 3.3637 and 3.3599 eV dominate the NBE emissions region at low temperature 10 K. The intensity of these recombinations decrease rapidly with increasing temperature, their position shifts slightly towards low energy and vanishes around 100 K. These modifications characterize the neutral donor-bound exciton D°X. In Fig. 14 we plot the curve of the observed energies of FX_A_ and of D°X as a function of the temperature. We assume that the peak positions of the two emissions FXA and D°X vary with temperature as for the energy band gap. Then, we have fit the energy temperature dependence curve with the Varshni's semi empirical formula:^17,49^16
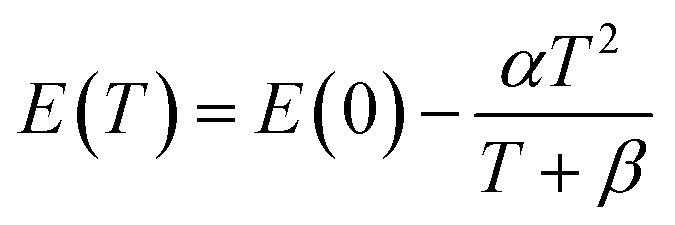


From Fig. 14, the Varshni curve is well compatible with the experimental values for the lines FX_A_ and D°X lines. The fitting parameters for FX_A_ and D°X lines are respectively:

*α* = 7.1382 10^−4^ eV K^−1^, *β* = 455 K and *E*(0) = 3.3795 eV and 3.3637 and 3.3599 eV.

The shallow zinc donor state involved in tetrahedral and octahedral interstitial sites proves the existence of two recombinations attributed to the neutral donor-bound exciton D°X (3.3637 and 3.3599 eV).

From the adjustment of the FX_A_ emission, we can conclude that the shift of this emission with increasing temperature can be due to the shrinking of the forbidden band induced by the temperature. The recombination of both FX_A_ and D°X at low temperatures is possible in the inter band excitonic transition.^50,51^ The thermal activation energy, with increasing the temperature (*T* > 60 K), is enough to a significant amount of D°X ionize into FX_A_(D°X → D° + FX_A_). Due to its high binding energy of 60 meV, FX_A_ will remain up to room temperature. The D°X emission is no longer visible in the PL spectrum. The emission spectra exhibited a prominent fluorescence peak at 368 nm in visible region due intrinsic and extrinsic defects. An excitonic line, labeled as the surface excitons (SX) for ZnO, is detected between the FXA and D°X lines at 3.3676 eV. The SX line's intensity is found to be lower than its nanoscale counter-part, which has been related to surface-volume ratio effects. The line positioned at 3.3107 eV at 10 K is maintained up to room temperature and becomes dominant at 300 K, where it is located at 3.2645 eV. This line has the same characteristics as the BF emission line reported in Co^2+^ doped ZnO NPs at 3.314 eV at 15 K and in Co^2+^ doped ZnO NPs at 3.3187 eV at low temperature 10 K, according to comparison.

The recombination of defect-bound carriers with free carriers in some bands is assigned to this line. The experimental data of the BF peak position at various temperatures fit well to the curve (dotted line in Fig. 14) calculated using the formula describing the peak position of emission due to transitions from a donor to the valence band:^52^

17 *hν* = *E*_g_ − *E*_D_ + *k*_B_*T*

*E*_g_ denotes the band-gap energy, *E*_D_ is the donor's binding energy, *T* denotes temperature, and *k*_B_ denotes the Boltzmann constant. According to Varshni's model, the position peak of the BF emission fluctuates slower with temperature than the energy band gap, as illustrated in Fig. 14.

At 10 K in Fig. 13, several additional peaks appear on the low energy side. These peaks are assigned to the longitudinal optical phonon replica (LO) of the donor bound excitons D°X and BF emission. This interpretation is based on the average spacing of 72 meV.

**Fig. 15:**
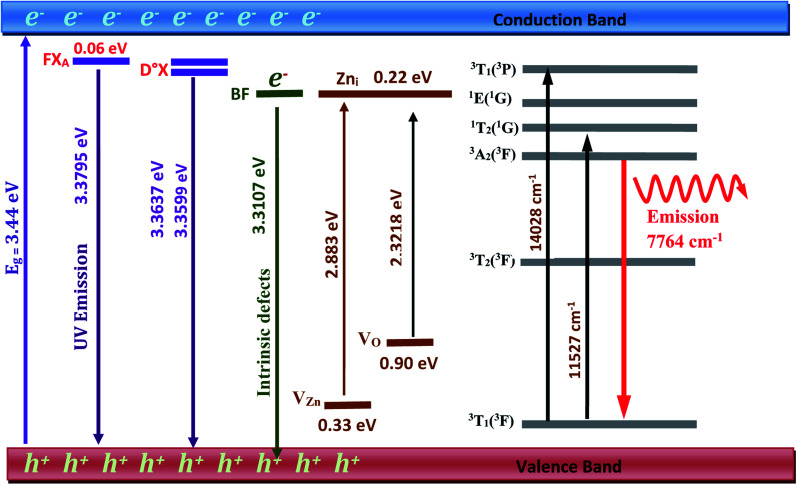
UV-Vis-near infrared energy level diagram of ZnO:Ni^2+^ NPs.

The peaks at 3.2922, 3.2211, and 3.1536 eV are thought to be D°X (D°X-1LO, D°X-2LO, and D°X-3LO) phonon replicas, the peaks at 3.2360 and 3.1662 eV are thought to be BF phonon replicas (BF-1LO and BF-2LO). Fig. 15 shows all the optical transitions demonstrated in this work.

## 5 Conclusion

Ni-doped ZnO NPs were successfully prepared by co-precipitation method. The structural and morphological properties of the synthesized nanoparticles have been studied using several techniques. EDX and XRD show a good crystallinity of prepared samples and the incorporation of Ni^2+^ in ZnO structure. It confirms a single phase with a hexagonal wurtzite structure of ZnO lattice (space group *P*6_3_*mc*). The crystallite size determined by the Williamson–Hall model is bigger than that obtained by the Scherrer model. The results of the Williamson–Hall model are more precise because the constraint *ε* is taken into account and the data is fitted according to this method. The grain size obtained from TEM is equivalent with crystallite size calculated by Willamson–Hall method. From TEM, the morphology of the ZnO:Ni NPs shows a hexagonal structure of spherical shape with smaller grain size compared to undoped ZnO. The SAED ring pattern suggests that the ZnO NPs phase was polycrystalline in structure, and the distance between crystalline planes was consistent with the standard pattern for a wurtzite ZnO crystal structure. FTIR spectra revealed the existence of all functional groups, and this study proved that Ni^2+^ is occupying the Zn^2+^ position. From the UV photoluminescence spectra, we interpret the ZnO near-band-edge NBE ultraviolet emission. The good fit with Varshni's semi empirical formula of the peak positions *versus* temperature confirms the two near-band-edge NBE ultraviolet emission: the free exciton FX_A_ and the donor bound exciton emission D°X. The observed values of the BF peak position at various temperatures fit well to the curve calculated using the formula describing the peak location of emission owing to transitions from a donor to the valence band. The blue–green absorption in ZnO NPs could be owing to an electron transition from the valence band to the Zn interstitial energy level, which is caused by defects. Another absorption in the green region is related to deep level absorption (DLE). It consists with the oxygen vacancies *V*_O_. From the theoretical crystal-field analysis and the visible region, the electronic structure of the Ni^2+^ doped zinc oxide nanoparticles is determined. The *T*_d_ site symmetry of Ni^2+^ in ZnO host crystal is confirmed in this theoretical work, which leads to excellent correlations between observed and computed energy levels. According to this study, Ni doping enhances the optical properties of ZnO nanoparticles. In addition to the ultraviolet near-band transitions and the visible blue–green transition, the incorporation of Ni^2+^ in ZnO semiconductor with a high gap leads to the near-infrared emission at 1187 nm which is attributed to intra-3d–3d transition ^3^A_2_(^3^F) → ^3^T_1_(^3^F). The d–d transitions of Ni^2+^ are located in the large gap of ZnO nanoparticles. For this reason, ZnO:Ni^2+^ can be used for optoelectronic devices such as near-infrared light-emitting diodes (N-IR LEDs). Moreover, Ni^2+^ doping increases the ZnO matrix defects, which increases the luminescence of the blue–green region.

## Conflicts of interest

There are no conflicts of interest to declare.

## Supplementary Material
